# Attrition of Nursing Professionals in Ghana: An Effect of Burnout on Intention to Quit

**DOI:** 10.1155/2022/3100344

**Published:** 2022-07-12

**Authors:** Douglas Aninng Opoku, Nana Kwame Ayisi-Boateng, Joseph Osarfo, Alhassan Sulemana, Aliyu Mohammed, Kathryn Spangenberg, Ali Baba Awini, Anthony Kwaku Edusei

**Affiliations:** ^1^Department of Occupational and Environmental Health, School of Public Health, Kwame Nkrumah University of Science and Technology, Kumasi, Ghana; ^2^Allen Clinic, Family Healthcare Services, Kumasi, Ghana; ^3^Department of Medicine, School of Medicine and Dentistry, Kwame Nkrumah University of Science and Technology, Kumasi, Ghana; ^4^University Hospital, Kwame Nkrumah University of Science and Technology, Kumasi, Ghana; ^5^Department of Community Medicine, School of Medicine, University of Health and Allied Health Science, Ho, Ghana; ^6^Department of Environmental Science, Kwame Nkrumah University of Science and Technology, Kumasi, Ghana; ^7^Department of Epidemiology and Biostatistics, School of Public Health, Kwame Nkrumah University of Science and Technology, Kumasi, Ghana; ^8^Family Medicine Directorate, Komfo Anokye Teaching Hospital, Kumasi, Ghana; ^9^Department of Health Promotion and Education, School of Public Health, Kwame Nkrumah University of Science and Technology, Kumasi, Ghana

## Abstract

**Background:**

Burnout among nursing professionals at the workplace and how it influences their decision to quit the profession is crucial to the delivery of quality health service. The shortage of nursing professionals has serious consequences on the healthcare system.

**Aim:**

To examine the effect of burnout on intention to quit the profession among nursing professionals.

**Methods:**

A cross-sectional study among 375 randomly selected nursing professionals in active service at a tertiary healthcare setting in Kumasi, Ghana. The Maslach Burnout Inventory was used to determine burnout, and their intention to quit the profession was assessed by asking participants whether they ever thought about quitting the profession in the past 12 months. The effect of burnout on intention to quit was analyzed using logistic regression analysis.

**Results:**

The overall prevalence of burnout among participants was 2.1% (8/375) with 10.1% (38/375), 24.0% (90/375), and 56.3% (211/375) experiencing high emotional exhaustion, depersonalisation, and low personal accomplishment, respectively. Nearly half (49.3%, 185/375) of the participants had intention to quit the profession. Emotional exhaustion (adjusted odds tatio, AOR = 5.46; 95% CI = 2.25–13.20), depersonalisation (AOR = 1.77 95% CI = 1.07–2.95), and personal accomplishment (AOR = 2.27; 95% CI = 1.30–3.96) were associated with intention to quit the profession.

**Conclusion:**

Burnout has a negative effect causing intention to quit nursing profession. It is imperative to identify strategies such as occupational health surveillance that will aim at reducing the incidence of burnout at the workplace due to its consequences, one of them being the intention to quit.

## 1. Background

Burnout and turnover intention among nurses and midwives are often studied phenomena in the healthcare settings and continue to be a major public health issue. Burnout, due to its severity on professionals, has been accepted as an occupational phenomenon by the World Health Organization's (WHO) International Classification of Diseases (ICD-11) [1]. Burnout is defined by the WHO as “a syndrome conceptualized as resulting from chronic workplace stress that has not been successfully managed [1].” The Action Collaborative on Clinician Well-Being and Resilience was launched by the National Academy of Medicine (United States) to address the increasing rate of burnout among healthcare professionals by improving the understanding of challenges to clinician well-being, advocating for clinician burnout, and prefer evidence-based, multidisciplinary solutions that will improve patient care by giving care to healthcare professionals [2]. Burnout has been classified into three dimensions: emotional exhaustion, depersonalisation, and lack of personal accomplishment [3]. Burnout directly affects the health of the professional (depression, suicide, substance use etc.) and adversely affects the healthcare system [4–6]. One such group of health professionals that easily suffer burnout are the nursing professionals. Burnout lowers nurses' quality of life, performance level, and organizational commitment and increases their intention to quit the job [7, 8]. Burnout also increases turnover rates and negatively affects the quality of nursing care [9, 10].

Studies that were conducted before the coronavirus disease (COVID-19) reported a high prevalence of burnout in the nursing profession similar to other healthcare professionals [7, 11, 12]. In Sweden, a study reported that approximately 50% of new nurses experienced burnout which influenced their intention to quit the profession [13]. Studies that were conducted during the outbreak of the COVID-19 reported a high prevalence of burnout and other psychological disorders such as moral injury, insomnia, anxiety, and depression among healthcare workers (HCWs) [14, 15]. As frontline health workers that were heavily involved in the case detection and management of COVID-19 and other illnesses, they were exposed to pressure. This thus resulted in a lot of them getting infected with the COVID-19 disease. In Italy, data released by the National Health Institute on 26 February 2021 showed that about 123,025 HCWs were infected with COVID-19 which was about 4.0% of the total cases since the outbreak of the pandemic [16]. About 152,000 and 1413 COVID-19 infections and mortality, respectively, were also reported among HCWs globally as of May 8, 2020 [17]. During the COVID-19 pandemic, it was reported that predictors such as being a female and a frontline HCW were significantly associated with burnout [14].

Burnout has been linked to nurses' intention to quit the profession [18, 19]. In Ghana, moderate to high burnout has been reported among nurses and other healthcare professionals [20–22]. Individuals have several mechanisms that they adopt to cope with high workloads or stress at the workplace. One can either continue to be exposed to these conditions for a long time and end up experiencing burnout or quit the profession or organization. Several determinants of burnout among nurses and midwives in Ghana, particularly high workload, years of practice, resilience, inadequate staffing, and lack of leadership support have been identified [21, 23]. Intention to quit the profession is one's desire or contemplation to quit the current profession in the future [24]. Up to 50% of nurse respondents have indicated their intention to quit the educational course in Italy, South Africa, and Ethiopia [25–27]. Similarly in Ghana, a prevalence rate of 69.0% of nurses with intention to quit their profession has been reported [28]. The predictors to understand intention to quit the profession in the nursing profession have been explored [28, 29].

Most of the studies that have been conducted either focused on only burnout [3, 7, 20] or intention to quit the profession [27, 29, 30]. Little is known about the two phenomena (burnout and intention to quit the profession) simultaneously, especially in the nursing profession in Ghana and Africa. This study, therefore, sought to determine the prevalence of intention to quit the profession and examine the effect of burnout on intention to quit the profession among nurses and midwives. The study findings are expected to feed into future policies to reduce the rate of burnout and intention to quit the profession among the nursing professionals in Ghana and ultimately in Africa. This is deemed key against the backdrop of the current COVID-19 pandemic and beyond.

## 2. Methods

### 2.1. Study Design

This was a cross-sectional study that involved nursing professionals at Komfo Anokye Teaching Hospital (KATH), a tertiary healthcare institution located in Kumasi, Ghana.

### 2.2. Study Setting and Population

Komfo Anokye Teaching Hospital has a bed capacity of 1,200. It serves as a referral centre for health facilities in the northern belt of the country. Komfo Anokye Teaching Hospital has about 2000 nursing professionals with different professional backgrounds.

The target population was all nursing professionals at the facility. Nursing professionals in this study were defined as registered nurses and midwives that provide nursing care to patients. Nursing professionals at the facility that had worked for at least one year were included in the study. All nursing professionals that were on their annual leave throughout the time of the study were excluded.

### 2.3. Sample Size Calculation

The Charan and Biswas [31] formula (sample size=(*Z*^2^(*P*)(1 − *P*))/(*E*^∧2^)  for calculating sample size was used to determine the sample size for this study. Using a 95% confidence interval (*Z*), 5% allowable margin of error E, and 64.9% [29] of nurses that had intention to quit (*P*), a sample size of 350 was obtained for the study. This was overestimated to 385 participants to cater for a nonresponse rate of 10%.

### 2.4. Data Collection and Sampling Procedure

Data collection took place from July 1, 2020 to September 31, 2020. This was done after the first wave of the COVID-19 pandemic in Ghana. A structured questionnaire that consisted of the Maslach Burnout Inventory (MBI) [3] was used to collect data from study participants. The MBI is a validated tool that has been adopted in several studies to assess burnout [32, 33]. The questionnaire was pretested among 15 nursing professionals in a government hospital in a nearby district.

The MBI tool measures the three burnout components (emotional exhaustion, depersonalisation, and personal accomplishment). The MBI is made up of 22 items (emotional exhaustion = 7 items, depersonalisation = 7 items, and personal accomplishment = 8 items) with all the items evaluated on a 7-point Likert scale ranging from “never” = 0 to “every day” = 6. The first seven items measure the emotional exhaustion dimension while the second seven items measure the depersonalisation dimension. The last eight items measure the personal accomplishment dimension of burnout. The burnout scores for each of the dimensions of burnout were computed and categorized as low, moderate, and high. The emotional exhaustion dimension was categorized as low (≤17), moderate (18–29), and high (≥30). The depersonalisation dimension was categorized as low (<6), moderate (6–11), and high (≥12: high) while the personal accomplishment dimension was categorized as low (≥40), moderate (34–39), and high (≤33). The Cronbach alpha's coefficient reliability test for this study was 0.773, 0.624, and 0.788 for emotional exhaustion, depersonalisation, and personal accomplishment, respectively. The overall burnout in this study was defined as the proportion of staff with high emotional exhaustion, depersonalisation, and low personal accomplishment.

The intention to quit the profession was measured by asking study participants if they had considered leaving the profession in the past 12 months. The question elicited a “yes” or “no” response with “yes” indicating that the participant had considered quitting the profession in the past 12 months. Participants that indicated they had considered quitting the profession were asked about reasons for their intention to quit the profession.

The simple random sampling technique was used to recruit 375 study participants from six (randomly selected) out of thirteen clinical departments. The total number of active nurses in each department was retrieved, and a proportional sample was estimated. Specifically, the total number of nurses in a selected department was divided by the total number of nurses in all the selected departments. A code was assigned to each nurse and these were written on a piece of paper and put in a bowl. This was shaken to ensure that they were evenly mixed. The papers were selected one after the other until the allocated number for each department was obtained. The participants that were recruited were all those who were approached by the investigators and agreed to participate in the study. The purpose of the study, risks, benefits, and confidentiality of the study were explained to all the study participants before being recruited into the study. All study participants that consented to take part in the study signed informed consent.

### 2.5. Data Management and Analysis

Data were entered into an excel spreadsheet. The data were cleaned and double-checked to ensure that there were no double and wrong entries. Data were exported to Stata version 16 for analysis and presented as means, frequencies, percentages tables, and graphs. The relationship between burnout and intention to quit was analyzed using the chi-square test and logistic regression analysis. A *p*-value of ≤0.05 was deemed statistically significant.

## 3. Results

### 3.1. Demographic and Work-Related Characteristics of Study Participants

Table 1 indicates the demographic and work-related characteristics of study participants. This study recorded a response rate of 98.7% (375/385). The mean age of the study participants was 31.5 (SD ± 5.0) with a minimum age of 21 years and a maximum age of 57 years. More than fifty per cent (56.0%, 210/375) were between the ages 30 and 39 years. Over eighty per cent (84.5%, 317/375) were females, and 54.4% (204/375) had diploma education.

Approximately 36.8% (138/375) of the study participants were of the rank “staff nurse/midwife.” The mean working hours per week was 41.8 hours with a range of 20 to 96 hours. Approximately 46.9% (176/375) of the participants worked for 31–40 hours per week. The median working experience of the study participants was 4.0 years with a range of 1 to 30 years. Approximately 56.0% (210/375) of the participants had a working experience of between 6 and 10 years. Over 82.4% (309/375) of the participants indicated their reason for working as a nurse/midwife was their passion to care for the sick.

### 3.2. Intention to Quit the Profession and Burnout among Study Participants

**Figure 1:**
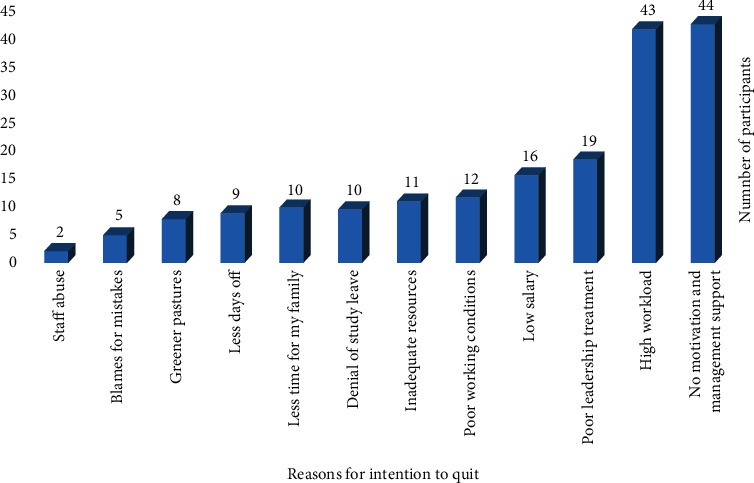
Reasons for intention to quit the profession among study participants.

The proportion of study participants that had an intention to quit the profession was 49.3% (185/375) (Table 2). Lack of motivation and management support (*n* = 44) was the predominant reason for having intention to quit the profession by study participants (Figure 1).

The overall prevalence of burnout among study participants was 2.1% (8/375). About 10.1% (38/375) of the study participants experienced high emotional exhaustion while over 24.0% (90/375) experienced high depersonalisation. About 56.3% (211/375) experienced low personal accomplishment (Table 2).

### 3.3. Relationship between Burnout and Intention to Quit the Profession

Table 3 represents the relationship between burnout and intention to quit among study participants. There was a statistically significant relationship between emotional exhaustion (*p* < 0.001), depersonalisation (*p* < 0.001), personal accomplishment (*p*=0.002), and intention to quit the profession.

Table 4 represents the multiple logistic regression analysis of the relationship between burnout and intention to quit among study participants. The odds of participants that experienced high emotional exhaustion having an intention to quit the profession was about five times (Adjusted Odds Ratio, AOR = 5.46; 95% CI = 2.25–13.20, *p* < 0.001) higher compared to those that experienced low emotional exhaustion. The odds of participants that experienced moderate depersonalisation having an intention to quit the profession was about two times (AOR = 1.77 95% CI = 1.07–2.95, *p*=0.027) higher compared to those that experienced low depersonalisation. Similarly, the odds of participants that experienced high personal accomplishment having an intention to quit the profession was about two times (AOR = 2.27; 95% CI = 1.30–3.96, *p*=0.004) higher compared to those that experienced low personal accomplishment.

## 4. Discussion

The study showed the prevelence of an overall burnout and intention to quit that is about 2.1% and 49.3%, respectively. All dimensions of burnout were found to be significantly associated with intention to quit the profession. To the best of our knowledge, this is the first study to report on the effect of all the dimensions of burnout on intention to quit the profession among nursing professionals in Ghana. The growing demand for quality healthcare services makes it essential to improve the human resource of the healthcare industry. Retention of nursing professionals is the key to sustaining and improving the health system and quality healthcare delivery. Reducing burnout among these professionals is a key step towards this goal. Identifying the effect of burnout on intention to quit the profession will help all key stakeholders of health to set up public health policies that will reduce the incidence of burnout and intention to quit the profession to the barest minimum at the workplace.

The outcome of the present study suggests that the overall prevalence of burnout among nursing professionals was low (2.1%). The prevalence of the dimensions of burnout in this study were 10.1% for high emotional exhaustion, 24.0% for high depersonalisation, and 56.3% for low personal accomplishment. Our study is similar to an earlier one that reported 10.8% high emotional exhaustion, 5.5% high depersonalisation, and 65.0% low personal accomplishment among health workers in Ghana [22]. The present study is also consistent with a similar study in China that found that about 8.02%, 15.93%, and 79.21% of nurses experienced high emotional exhaustion, depersonalisation, and low personal accomplishment, respectively, during the COVID-19 outbreak. However, our findings are at variance with those from a study that reported a pooled prevalence of high burnout of 51.0% emotional exhaustion, 52.0% depersonalisation, and 28% low personal accomplishment with about 52.0% of healthcare workers experiencing overall burnout during the COVID-19 pandemic [34]. In the United States, 65.1% high emotional exhaustion, 38.40% high depersonalisation, and 90.4% low depersonalisation have been reported among nurses working in a tertiary healthcare centre [35]. The variations in the study could be attributed to the different study designs and the severity of the COVID-19 pandemic. The impact of the COVID-19 pandemic was very severe in the United States which could influence the nurses in the United States experiencing high burnout compared to the nurses in this study. The study reporting an overall burnout prevalence of 52.0% among HCWs [35] was a systematic review and meta-analysis that assessed a pooled prevalence of burnout which could increase the burnout rates compared to this study.

In this study, nearly half of the study participants had intentions to quit the profession. This is a matter of concern, especially amidst a global pandemic. This can also be very challenging for the health system as a whole where nursing professionals require optimum motivation and needed resources to deliver quality healthcare to patients. The participants harbouring an intention to quit the profession can result in high turnover among the nursing professionals which can affect quality healthcare delivery. This is because those intending to quit the profession have been in practice for at least four years and have acquired some experience which will be difficult to replace in a short time.

The proportion of participants intending to quit the profession in the present study (49.3%) is comparable to other reports of 46.1% in South Africa [25] and 44.6% in Japan [30]. However, it is higher than the findings of a multicentre European study in which a prevalence of 9.0% was reported among nurses [36] and lower than the 64.9% reported in Ethiopia [29]. The variations in the study findings may be attributed to the study population, facility type, methods, and tools for measuring intention to quit the profession. In the Ethiopian study [29], intention to quit the profession was measured using the Hand tool [37] which measures intention to quit using seven questions with a 5-point Likert scale while the present study measured intention to quit with a single question that elicited a “yes” or “no” response.

In the current study, all the dimensions of burnout were associated with intention to quit. This is in line with other studies that reported that burnout affects intention to quit the profession among nurses and midwives [38–40]. The participants that experienced high burnout for emotional exhaustion and personal accomplishment were more likely to have an intention to quit compared to those that experienced low burnout for emotional exhaustion and personal accomplishment, respectively. This is in consonance with a study that reported that a feeling of high emotional exhaustion for a prolonged time may increase the thought of intention to quit the profession [26]. The effect of high burnout for personal accomplishment on intention to quit could be that after long years of practice, they may have given up challenging the unfavourable work conditions (such as poor leadership style, inadequate personal protective equipment, high workload etc.) since previous attempts may not have yielded fruit. This can influence their decision to consider quitting their job or changing the work environment.

Moderate depersonalisation was also found to increase intention to quit the profession by 77% in the multivariate regression. This is comparable to a study that found depersonalisation as a predictor of intention to quit the profession among nurses [41]. This could be because people that experience moderate to high burnout for depersonalisation may develop a negative attitude towards patients and work which can negatively affect their involvement in the workplace [42]. Developing a negative attitude towards patients and work can lead to the HCW feeling that his or her current profession may not be good for him or her. This can result in professional detachment in him or her from the work which could induce his or her decision to quit.

The outcome of this study highlights the need to pay critical attention to all the dimensions of burnout in an attempt to reduce intention to quit the profession among nursing professionals. It also highlights the need to improve on the personal accomplishment of the nursing staff for a high retention rate. It is therefore important for organizational managers to put in place interventions (such as capacity building and rewarding hard work) that will promote the feeling of personal accomplishment of nurses and midwives at the workplace. Nurse and organizational managers should put in place strategies such as reducing workloads by employing more nurses or providing adequate resources to work with.

It is important that in formulating policies to address the retention of nursing professionals, a premium should be placed on the psychological and mental health of the professionals such as reducing the incidence of burnout at the workplace due to its consequences on intention to quit and retention. This can be mitigated through regular occupational health surveillance and workplace health promotion programs implemented with strategies combined with interventions by public and occupational health stakeholders [43, 44]. In situations where these strategies are not efficient, burnout levels could increase and lead to higher intention to quit, cause reduction in the quality of care, and result in high malpractice litigation [45].

### 4.1. Limitation of the Study

Due to the use of cross-sectional study design, drawing statistical associations to causal effect between burnout and intention to quit the profession is limited. In addition, the study findings are of limited generalizability as the study was conducted in only one facility. The odds ratio estimate for the association between high emotional exhaustion and intention to quit had a rather wide confidence interval suggesting low precision and should be interpreted with caution. The use of a structured questionnaire did not allow for a qualitative exploration of participants' response choices and this limits full comprehension of the dynamics underlying burnout and intention to quit. Nevertheless, the study provides useful insights into burnout and intention to quit the profession among the study participants, and this can be beneficial for nurses and organizational managers.

## 5. Conclusion

Moderate to high burnout for all three dimensions increases the risk of intention to quit the profession among nursing staff at a tertiary healthcare facility in Ghana. Work-based programmes and strategies such as occupational health surveillance that aim at reducing the incidence of burnout can impact positively on intention to quit the profession among nursing professionals. Future studies should therefore adopt a qualitative approach to fully explore the dynamics of burnout and its effect on intention to quit the profession among nursing professionals.

## Figures and Tables

**Table 1:** Demographic and work-related characteristics of study participants.

Variables	Frequency	Percentage, % [range]
Age (years)		
>30	146	38.9
30–39	210	56.0
40+	19	5.1
Mean (±SD)	31.5 (±5.0)	[21–57]

Gender		
Male	58	15.5
Female	317	84.5

Marital status		
Married	212	56.5
Single	163	43.5

Religion		
Christian	355	94.7
Muslim	20	5.3

Level of education		
Diploma	204	54.4
Graduate	151	40.3
Post-graduate	20	5.3

Professional rank		
Staff nurse/midwife	138	36.8
Senior staff nurse/midwife	70	18.7
Nursing/midwifery officer	102	27.2
Senior nursing/midwifery officer	45	12.0
Principal nursing/midwifery officer	20	5.3

Reasons for working as a nurse/midwife		
Passion to care for the sick	309	82.4
To earn an income	18	4.8
The program I chanced on after school	4	1.1
Not indicated	44	11.7

Working experience (years)		
1–5	146	38.9
6–10	210	56.0
11+	19	5.1
Median (years)	4.0	[1–30]

Working hours per week (hours)		
<31	26	6.9
31–40	176	46.9
41–50	154	41.1
51+	19	5.1
Mean (±SD)	41.8 (±8.5)	[20–96]

SD: standard deviation, not indicated = missing values, post-graduate: masters holders.

**Table 2:** Intention to quit the profession and burnout among participants.

Variables	Frequency	Percentage, % [range]
Intention to quit the profession		
Yes	185	49.3
No	190	50.7

Burnout emotional exhaustion		
Low	223	59.5
Moderate	114	30.4
High	38	10.1
Mean (±SD)	16.4 (±9.1)	[0–42]

Depersonalisation		
Low	143	38.1
Moderate	142	37.9
High	90	24.0
Mean (±SD)	8.1 (±6.1)	[0–41]

Personal accomplishment		
Low	211	56.3
Moderate	80	21.3
High	84	22.4
Mean (±SD)	38.4 (±8.5)	[2–48]

Overall burnout		
Yes	8	2.1
No	367	97.9

SD: standard deviation.

**Table 3:** Relationship between burnout and intention to quit among study participants.

Variable	Intention to quit		*p*-value
	Yes *n* (%)	No *n* (%)	
Emotional exhaustion			<0.001
Low	84 (37.7)	139 (62.3)	
Moderate	71 (62.3)	43 (37.7)	
High	30 (79.0)	8 (21.1)	

Depersonalisation			<0.001
Low	52 (36.4)	91 (63.6)	
Moderate	83 (58.5)	59 (41.6)	
High	50 (55.6)	40 (44.4)	

Personal accomplishment^*∗*^			0.002
Low	88 (41.7)	123 (58.3)	
Moderate	44 (55.0)	36 (45.0)	
High	53 (63.1)	31 (36.9)	

**Table 4:** Multiple logistic regression analysis of the relationship between burnout and intention to quit among study participants.

Variables	Unadjusted OR (95% CI)	*p*-value	Adjusted OR (95% CI)	*p*-value
Emotional exhaustion				
Low	1.00		1.00	
Moderate	2.73 (1.72–4.35)	<0.001	2.49 (1.51–4.09)	<0.001
High	6.21 (2.72–14.17)	<0.001	5.46 (2.25–13.20)	<0.001

Depersonalisation				
Low	1.00		1.00	
Moderate	2.46 (1.53–3.97)	<0.001	1.77 (1.07–2.95)	0.027
High	2.19 (1.28–3.74)	0.004	1.06 (0.57–1.98)	0.843

Personal accomplishment				
Low	1.00		1.00	
Moderate	1.71 (1.02–2.87)	0.043	1.52 (0.88–2.62)	0.135
High	2.39 (1.42–4.02)	0.001	2.27 (1.30–3.96)	0.004

NB: each of the variables emotional exhaustion, depersonalisation, and personal accomplishment was adjusted for the other two ORs (odds ratio)s.

## Data Availability

The datasets used for this study are available from the corresponding author upon reasonable request.
